# Response to Therapeutic Plasma Exchange as a Rescue Treatment in Clinically Isolated Syndromes and Acute Worsening of Multiple Sclerosis: A Retrospective Analysis of 90 Patients

**DOI:** 10.1371/journal.pone.0134583

**Published:** 2015-08-05

**Authors:** Johannes Ehler, Sebastian Koball, Martin Sauer, Steffen Mitzner, Heiko Hickstein, Reiner Benecke, Uwe K. Zettl

**Affiliations:** 1 Department of Anaesthesiology and Intensive Care Medicine, University of Rostock, Rostock, Germany; 2 Department of Neurology, Neuroimmunology Section, University of Rostock, Rostock, Germany; 3 Department of Internal Medicine, Division of Nephrology, University of Rostock, Rostock, Germany; 4 KfH Dialysis Centre Wismar, Wismar, Germany; Charite—Universitätsmedizin Berlin, GERMANY

## Abstract

**Objectives:**

Experience with therapeutic plasma exchange (TPE) for acute relapses in clinically isolated syndrome (CIS) or multiple sclerosis (MS) patients has been derived from small and inhomogeneous patient populations so far. In the present study, we retrospectively evaluated features associated with TPE response in a larger cohort of CIS and MS patients with acute worsening of disease.

**Participants:**

Ninety CIS and MS patients with acute relapses or acute worsening of symptoms were firstly treated with TPE. The population consisted of 62 women and 28 men with a median age of 38 years (range 18–69 years).

**Outcome Measures:**

Primary endpoint was the clinical response to TPE, focused on the functional improvement of the target neurologic deficit. Secondary endpoint was an improvement in expanded disability status scale (EDSS) scoring.

**Results:**

A clinical response to TPE was observed in 65 out of 90 patients (72.2%), with marked improvement in 18 (20.0%) and moderate improvement in 47 out of 90 patients (52.2%). The median EDSS was reduced from 3.75 before to 3.0 after TPE (*p* = 0.001). Response to TPE was significantly more frequent in patients with relapsing courses of disease (CIS, RR-MS, *p* = 0.001), no disease modifying drugs (*p* = 0.017), gadolinium-positive (Gd^+^) MRI lesions (*p* = 0.001) and EDSS ≤ 5.0 before TPE (*p* = 0.014). In the multiple logistic regression analysis only the detection of Gd^+^ MRI lesions was significantly altered (*p* = 0.004).

**Conclusion:**

Clinical response to TPE was achieved in the majority of our patients. We identified clinical and diagnostic features in CIS and MS relapses that might be helpful to identify patients responding to TPE. Gd^+^ MRI lesions before treatment were the best predictor of the response to TPE in our cohort.

## Introduction

Multiple sclerosis (MS) is an immune mediated disease of the central nervous system (CNS) significantly causing proceeding disability in young adults [1–3]. The underlying mechanisms of this relapsing and often chronic progressive disease are insufficiently understood [4, 5]. Modern immunomodulating strategies, however, offer several therapeutic approaches to clinically isolated syndrome (CIS) and MS patients today [6–8]. First line treatment of acute relapses is high dose glucocorticosteroid (GCS) pulse treatment with initially 1g methylprednisolone (MP), given daily over 3–5 days. A higher second GCS pulse with up to 2g MP can be considered in unresponsive patients after an interval of 2 weeks [9–12]. If symptoms persist despite GCS treatment, the relapse is defined as GCS-unresponsive and therapeutic plasma exchange (TPE) is recommended [7, 9, 10, 13]. Meanwhile, TPE is implemented into therapeutic guidelines of a broad spectrum of neurological disorders [7, 14–17]. The elimination of humoral factors (antibodies, complement factors, cytokines and immune complexes), all assumed to be involved in inflammation and demyelination in CIS and MS, is currently regarded as the rationale for TPE [7, 18, 19]. Beneficial TPE effects have been detected in about 40–90% of patients with acute relapses [14, 16, 20–25]. Male sex, preserved reflexes and an early initiation of treatment were associated with successful TPE [22, 23, 25, 26]. The response to TPE in the majority of pre-existing inhomogeneous studies was derived from patients with optic neuritis (ON), neuromyelitis optica (NMO), MS and CIS [15–17, 22, 23, 25, 26]. Studies focusing on MS or CIS included predominantly small patient numbers of 4–60 patients each [14–16, 20–27].

Therefore, the aim of our study was to analyse the response to TPE in a larger population consisting of 90 CIS and MS patients.

## Materials and Methods

### Participants and inclusion criteria

Based on the 2001 Mc Donald criteria [28], TPE data were evaluated from 90 patients diagnosed with acute worsening of CIS or MS from February 2001 to June 2013. The study was approved by the local ethics board at Rostock University (identifier A 2015–0065). All patient information and records were anonymised and de-identified prior to analysis. Of 11 included CIS patients treated between 2001 and 2012, data were previously analysed and published recently [14].

Inclusion criteria were unresponsiveness to GCS treatment or pre-existing contraindications for the use of GCS and no previous TPE in the patients’ history [9, 10]. Deteriorated, unchanged and insufficiently improved symptoms (slight change in symptom without impact on function) after GCS treatment were defined as GCS-unresponsiveness. An acute relapse was defined as a new and definite clinical attack. According to this definition secondary-progressive (SP)-MS patients with superimposed relapses were included. Primary-progressive (PP)-MS patients with a clinical worsening of neurologic function were evaluated as well.

### Outcome measures

The Expanded Disability Status Scale (EDSS) was used for clinical evaluation and scoring before, during and after GCS treatment and TPE by EDSS-certified neurologists [29]. The response to treatment was analysed at the end of each patient’s final TPE session. Follow-up examinations were performed in our in- or outpatient department. Routinely, the interval to the clinical re-evaluation after TPE was 3 months at our centre.

#### a) Primary endpoint of the response to TPE

Definition of the response to treatment was primarily based on changes of the predominant neurological symptom (target neurologic deficit, assigned to a functional system) by clinician’s examination and by patient’s notification [17, 22, 30, 31]. Marked improvement was defined as clinically significant improvement in function. Moderate improvement represented a definite change of the neurologic deficit without significant impact on function within the functional score. No effect comprised unchanged symptoms. Deterioration represented patients with worsened target neurologic deficit or new neurologic symptoms.

#### b) Secondary endpoint of the response to TPE

Additionally, EDSS change during treatment was used to measure GCS effects and the response to TPE. Response was defined as an EDSS decrease ≥1.0 points in patients with an initial EDSS ≤5.5 or an EDSS decrease ≥0.5 points in patients with an initial EDSS ≥6.0 [32].

### Diagnostic procedures before TPE

Available data from MRI and motor-, somatosensory- and visual-evoked potentials (MEP, SSEP and VEP, respectively), were obtained employing standard diagnostic methods according to CIS and MS guidelines before the initiation of GCS treatment [28, 33]. The results are summarised in Table A in S1 File.

### TPE procedures

All 90 patients gave written informed consent for TPE and central venous access before the initiation of treatment. The plasma volume was estimated using nomograms [34]. A minimum of 3 TPE sessions per patient was determined with an interval of 2 days between procedures. Based on experience and kinetic considerations at least 3 TPE sessions should be performed for a reduction of circulating IgG of up to 70% [25, 35]. In severely affected patients, the first 2 TPE sessions were performed daily. Further treatments up to a maximum of 8 TPE sessions (2 days between procedures) were performed, if either none or only mild positive changes of the patient’s predominant neurologic symptom were seen. In every treatment session, a single plasma volume was exchanged (2.8 L in median, range 1.9–4.0 L). One litre of the replacement solution contained 750 mL Ringer lactate solution according to Hartmann (Ringer-Lactat nach Hartmann, B. Braun Melsungen AG, Melsungen, Germany) and 250 mL of human serum albumin 20% (Alburex 20%, CLS Behring GmbH, Marburg, Germany). The albumin concentration in the ready to use fluid was 5%. Patients with known coagulation disorders or patients with a high bleeding risk were treated with fresh frozen plasma instead of albumin solution. TPE procedures were performed via peripheral veins in 31 patients and via central venous access in 55 out of 90 patients. In 4 patients vascular accesses were switched from peripheral veins to central venous access due to failed vein puncture.

Different machine settings (platforms) were used for TPE (Fresenius Multifiltrate with plasma filter PSU2S, Fresenius Medical Care AG, Bad Homburg, Germany; Baxter BM 11/14, Baxter Healthcare, Deerfield, USA with Gambro PF1000 plasma filter, Gambro AB, Lund, Sweden; Miltenyi Life18 system with therapeutic plasma exchange set, Miltenyi Biotech, Bergisch-Gladbach, Germany). The inlet flow rate was 80–100 mL/min and the average plasma flow rate was 20–25 mL/min in treatments with peripheral vascular access. In treatments with central venous access the inlet flow rate was 150 mL/min with an average plasma flow rate of up to 35 mL/min.

### Statistical analysis

Univariate comparison of baseline characteristics concerning the response to TPE was performed using Pearson’s Chi-Square test for independent variables.

Comparison of continuous variables of EDSS values before and after TPE as well as at time of follow-up was performed using non-parametric tests (Wilcoxon-Test). Multiple logistic regression analysis (stepwise forward) was used to assess the effect of baseline and treatment variables on TPE response (disease modifying drugs (DMD) before TPE, number of previous relapses, time between GCS and TPE treatment, cumulative GCS dosage, symptom worsening during GCS treatment, EDSS before TPE, time to TPE, gadolinium-positive (Gd^+^) lesions in MRI). Statistical significance was indicated by *p* < 0.05 (IBM SPSS Statistics, Version 20, Chicago, IL, USA).

## Results

### Clinical characteristics and diagnostic findings before the initiation of TPE

Between February 2001 and June 2013 a total of 90 CIS and MS patients were treated with TPE. The patient population consisted of 62 female (68.9%) and 28 male patients (31.1%) and comprised patients with CIS, relapsing-remitting MS (RR-MS), SP-MS with superimposed relapses and PP-MS with acute worsening of disease (Table 1).

**Table 1:** Patient characteristics.

Characteristics	CIS	RR-MS	SP-MS	PP-MS	All patients
No. of patients	21	46	18	5	90
Age (yrs)^a^	32.0 (20–63)	37.5 (18–56)	42.0 (23–69)	47.0 (24–66)	38.0 (18–69)
Body weight (kg)^a^	75.0 (50–109)	67.5 (47–128)	65.0 (49–106)	70.0 (50–95)	69.5 (47–128)
Disease duration (mo)^a^	2.0 (1–5)	56.5 (1–273)	171.0 (3–249)	37.0 (10–158)	47.0 (1–273)
DMD (%)	0	60.9	83.3	60.0	51.1
Previous relapses^a^	1.0 (1)	3.0 (1–20)	10.0 (2–30)	3.0 (1–5)	3.0 (1–30)
Relapses per year^a^	1.0 (0.5–2.0)	1.0 (0–4)	0.9 (0.2–9.0)	1.0 (0.4–3.0)	1.0 (0–9)

CIS = clinically isolated syndrome, DMD = disease modifying drug, MS = multiple sclerosis, PP-MS = primary progressive MS with acute worsening, RR-MS = relapsing-remitting MS, SP-MS = secondary progressive MS with superimposed relapse.

^a^ Median (range).

A single functional system (monosymptomatic relapse) was affected in 15 out of 90 patients (16.7%) and multiple functional systems (polysymptomatic relapse) in 75 out of 90 patients (83.3%). In polysymptomatic relapse patients the predominant neurological symptom (target neurological deficit) was determined and assigned to the respective functional system to analyse the response to TPE. Out of 90 patients the affected functional systems were: visual function in 10, brainstem function in 10, sensoric function in 10, myelitis in 17, cerebellar function in 16, pyramidal function in 24 and mental function in 3 patients, respectively. In 28 out 90 patients (31.1%) a symptom progression within a functional system or an extension to other functional systems was observed during GCS treatment. DMD before TPE were present in 47 out of 90 patients (52.2%, Table B in S1 File). Details of baseline characteristics and diagnostic findings are shown in Tables A and B in S1 File.

### GCS treatment before the initiation of TPE

**Fig 1:**
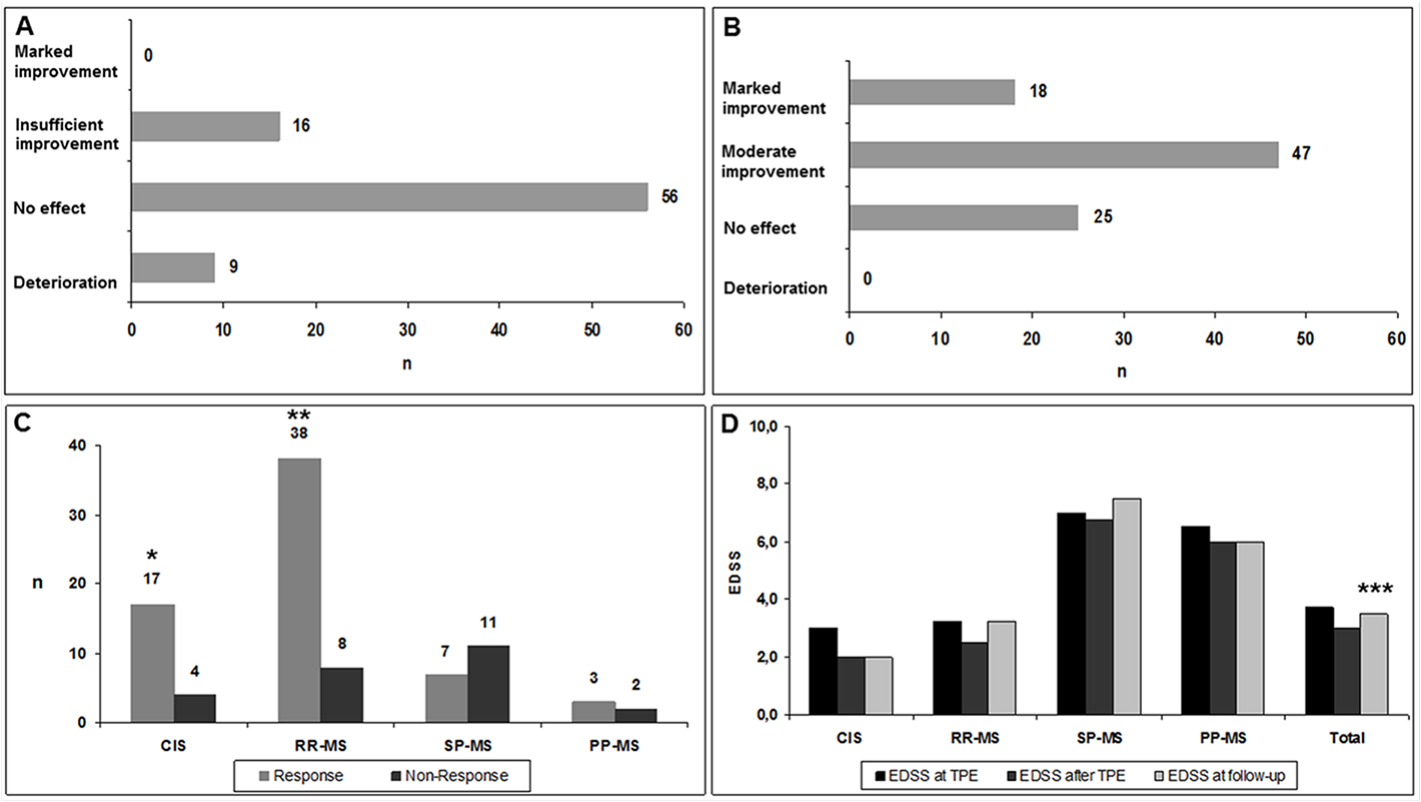
Glucocorticosteroid treatment and therapeutic plasma exchange in clinically isolated syndrome and multiple sclerosis patients. A) Response to glucocorticosteroid treatment in CIS and MS patients (n = 81). B) Response to therapeutic plasma exchange in CIS and MS patients (n = 90). C) Different responses to therapeutic plasma exchange in CIS and MS patients (n = 90). D) Development of median EDSS values during therapeutic plasma exchange (n = 90). CIS = clinically isolated syndrome, Deterioration = clinical symptom worsened and/or additional symptoms, Insufficient improvement = slight change in symptom without impact on function, Marked improvement = clinically significant improvement in function, Moderate improvement = definite change of the neurologic deficit without significant impact on function within the functional score, MS = multiple sclerosis, n = number of patients, No effect = clinical symptom unchanged, PP-MS = primary-progressive MS with acute worsening, RR-MS = relapsing-remitting MS, SP-MS = secondary-progressive MS with superimposed relapse, * *p* = 0.01 (CIS versus SP-MS), ** *p* = 0.002 (RR-MS versus SP-MS), *** *p* = 0.001 (EDSS before versus after TPE).

Eighty-one out of 90 patients received a high dose GCS treatment prior to TPE (Fig 1A). In 9 out of 90 patients, GCS were not administered to treat the acute relapse due to documented GCS-non-response during previous relapse treatment (n = 4) and due to known severe adverse events (steroid-induced pancreatitis, femoral head osteonecrosis with fracture) against GCS (n = 2). In three patients with an intermittent GCS treatment (1g MP given daily over 5 days every 3 months) the new clinical attack was evaluated as GCS-unresponsive and these patients immediately received TPE.

The median EDSS before GCS treatment was 3.75 (range 1.0–8.5). Median duration from the beginning of symptoms to the start of GCS treatment was 5.0 days (range 1–61 days). In 55 out of 81 patients (67.9%) GCS treatment comprised a pulse with 1 g MP/die and a following pulse with 2 g MP/die (ultra-high dose GCS treatment). Twenty-six out of 81 patients (32.1%) were treated merely with a pulse of 1 g MP/die due to rapid disease progression or adverse events related to GCS. The median cumulative dosage of GCS was 15.0 g MP per patient (range 3–15 g). Fifteen out of 81 patients (18.5%) complained adverse events during GCS treatment (gastrointestinal in 5, dermatologic in 3, cardiovascular in 3 and psychiatric in 4 patients, respectively).

### Response to TPE

All 90 patients received an initial TPE-series (Fig 1B–1D, Table 2). Median time from the beginning of symptoms to the start of TPE (time to TPE) was 57.5 days (range 2–163 days).

**Table 2:** Therapeutic plasma exchange in clinically isolated syndrome and multiple sclerosis patients.

	CIS	RR-MS	SP-MS	PP-MS	All patients
No. of patients	21	46	18	5	90
Time to TPE (days)^a^	50 (2–145)	60.5 (7–154)	61.0 (18–163)	99.0 (7–152)	57.5 (2–163)
No. of TPE^a^	6 (2–8)	5 (3–8)	5 (2–8)	5 (3–5)	5 (2–8)
TPE until effect^a^	5 (2–8)	5 (2–8)	5 (2–7)	3 (3–5)	5 (2–8)
Clinical response to TPE^a^	17 (81%)	38 (82.6%)	7 (38.9%)	3 (60.0%)	65 (72.2%)
EDSS before TPE^a^	3.0 (1.0–8.5)	3.25 (1.0–6.5)	7.0 (5.5–8.5)	6.5 (3.0–7.5)	3.75 (1.0–8.5)
EDSS after TPE^a^	2.0 (0.0–8.0)	2.5 (1.0–6.5)	6.75 (5.5–8.5)	6.0 (2.5–7.5)	3.0 (0.0–8.5)
EDSS at follow-up^a^	2.0 (0.0–7.5)	3.25 (1.0–7.0)	7.5 (4.5–8.5)	6.0 (3.0–8.0)	3.5 (0.0–8.5)

CIS = clinically isolated syndrome, EDSS = expanded disability status scale, MS = multiple sclerosis, No. = number, PP-MS = primary progressive MS with acute worsening, RR-MS = relapsing-remitting MS, SP-MS = secondary progressive MS with superimposed relapse, TPE = therapeutic plasma exchange.

^a^ Median (range).

Based on our clinical response definition, responses to TPE were observed in 65 out 90 patients (72.2%). Marked improvement were seen in 18 (20.0%) and moderate improvement in 47 out of 90 patients (52.2%). The response to TPE was documented after a median of 5.0 single TPE sessions (range 2–8 sessions). Twenty-five out of 90 patients (27.8%) were non-responder to TPE. Regarding our EDSS response definition, 31 out 90 patients (34.4%) were responder and 59 out of 90 patients (65.6%) were unresponsive to TPE. A significant EDSS decrease (*p* = 0.001) between median EDSS values before (3.75, range 1.0–8.5) and after TPE (3.0, range 0.0–8.5) was observed (Fig 1D, Table 2).

### Analysis of TPE-efficiency

All clinical and diagnostic baseline variables were analysed regarding to the response to TPE. In the univariate statistical analysis, patients without a pre-existing medication of DMD (*p* = 0.017) and without worsening during GCS treatment (*p* = 0.043) showed a significant response to TPE. Beneficial TPE was significantly more frequent in CIS (*p* = 0.010) and RR-MS (*p* = 0.002) patients as in SP-MS patients. In direct comparison of relapsing (CIS, RR-MS) and progressive disease (SP-MS, PP-MS), response to TPE was significantly more frequently observed in relapsing courses (*p* = 0.001). Moreover, response to TPE was significantly more often observed in patients with a baseline EDSS ≤ 5.0 (*p* = 0.014). In contrast to T_2_ lesions in MRI, the detection of Gd^+^ lesions was strongly associated with the response to TPE (*p* = 0.001). Gender, age, number of previous relapses, time to TPE, affected functional systems and pathological evoked potential results were independent from the response to TPE. In addition, there was no significant difference between patients treated with a pulse of 1 g MP/die and patients treated with 1g and 2g MP/die before TPE (Table C in S1 File).

Based on the completely available data in 70 of our patients (MRI exams were not available in 13 and a cumulative MP dose in 9 patients; 2 of these patients had neither MRI nor MP), a multiple logistic regression model was conducted. Gd^+^ lesions in MRI (*p* = 0.004) emerged as the only factor predicting the response to TPE in our patients (Table 3).

**Table 3:** Multiple logistic regression analysis of clinical baseline variables in clinically isolated syndrome and multiple sclerosis patients (n = 70).

Independent variable	OR	95% CI	*p*
Time to TPE	0.994	0.977–1.011	0.485
DMD before TPE	0.419	0.085–2.072	0.286
**Gd** ^**+**^ **lesions in MRI before TPE**	**0.095**	**0.019–0.479**	**0.004**
Number of relapses before TPE	1.093	0.915–1.306	0.325
EDSS before TPE	0.95	0.616–1.466	0.815
Cumulative MP dose before TPE	0.647	0.259–1.617	0.352
Time to GCS treatment before TPE	1.042	0.995–1.092	0.082
Worsening during GCS treatment	0.391	0.066–2.325	0.302

CIS = clinically isolated syndrome, DMD = disease modifying drug, EDSS = expanded disability status scale, GCS = glucocorticosteroid, Gd^+^ = gadolineum contrast medium enhancement, MP = methylprednisolone, MRI = magnetic resonance imaging, MS = multiple sclerosis, TPE = therapeutic plasma exchange.

### Adverse events of TPE

In 25 out of 466 single TPE-procedures (5.4%) adverse events occurred in 23 out of 90 patients (25.6%, 2 patients had two adverse events each). None of our patients died during the observation time. Severe adverse events were allergic reactions during TPE in three patients (mild allergic reaction to fresh frozen plasma in 1 patient, allergy to albumin in 1 patient, unknown cause of allergy in 1 patient, respectively) and a systemic infection with septic shock after the third TPE session in 1 patient. For this case, TPE was interrupted for intensive care treatment and was finished after the recovery. In 1 case with symptomatic segmental pulmonary embolism during the second session, TPE was interrupted for the treatment with low molecular heparin (Table 4). In 14 out of 67 patients (20.9%) with relapsing disease courses (CIS, RR-MS), adverse events occurred. In patients with progressive disease courses (SP-MS, PP-MS), adverse events were detected in 4 out of 23 patients (17.4%). The differences between relapsing and progressive disease groups were not significant (*p* = 0.488).

**Table 4:** Adverse events during therapeutic plasma exchange (n = 466 single sessions).

Adverse event	No.	Treatment
Local infection due to vascular access	1	Local antiseptics
Local bleeding due to vascular access	3	Local compression
Pain due to vascular access	1	Peripheral analgetics
Electrolyte imbalance (hypocalcaemia)	1	10% calcium gluconate
Temporary paresthesia	1	No treatment
Allergic reaction	3	Antihistamines + Prednisolone
Moderate hypotension	2	Crystalloid infusion
Coagulation imbalance	4	Specific substitution
Dislocation of peripheral vascular access	1	Change of access
Failed puncture for central venous access	1	No treatment
Vasovagal syncope	1	Symptomatic treatment
Systemic infection without sepsis	4	Antibiotics
Systemic infection with sepsis	1	Sepsis treatment on ICU
Thrombosis and pulmonary embolism	1	Anticoagulation
**Total adverse events**	**25**	

ICU = intensive care unit, Moderate hypotension = systolic blood pressure below 95 mmHg, No. = number.

### Follow-up examination and further relapses after TPE

Eighty-seven out of 90 patients (96.7%) could be subjected to follow-up examinations after TPE at our MS centre. With a median time of 62 days (range 9–353 days) after their final TPE session, patients were re-evaluated for clinical and EDSS scoring (median EDSS at follow-up 3.5, range 0.0–8.5). The comparison of EDSS values at the end of the final TPE session (EDSS 3.0) and at the time of follow-up (EDSS 3.5) showed a non-significant increase in EDSS (*p* = 0.256). In 35 out of 87 patients (40.2%) a new relapse was observed during long-term follow-up (observation deadline: June 2013). The median time to relapse after their last TPE session was 101 days (range 4–2487 days). The median EDSS was 4.5 (range 1.0–9.0).

At the end of long-term follow-up, 2 patients were diagnosed with CIS (2.3%), 58 with RR-MS (66.7%), 20 patients with SP-MS (23.0%), 6 with PP-MS (6.9%) and 1 patient with NMO (1.2%).

## Discussion

Previous TPE studies [16, 17, 20, 22] focused on acute exacerbations of different CNS inflammatory diseases (CIS, MS, ON or NMO). Therefore, clinical experience was derived from inhomogeneous patient populations. Pre-existing studies predominantly included a small number of patients [14, 16, 17, 20–22]. Current prospective clinical trials focusing on TPE in CIS and MS are non-existent. Hence, systematic evaluations of clinical variables and its associations with the response to TPE are of interest for daily clinical routine.

The results of TPE from our study were derived from a population of 90 patients with MS spectrum disorders (CIS and MS), the great majority of them was GCS-unresponsive. Our finding of about 72% of responders to TPE corresponds with previous reports and could supplement the pre-existing literature with new aspects [22, 23, 25].

In order to measure response to TPE accurately, we primarily focused on the changes in a target neurological deficit and secondarily in the EDSS. Quantitative EDSS rating is supposed to be favourable in comparison to subjective clinical examination of single symptoms. Otherwise, pure evaluation of EDSS values is limited by insufficient symptom representation in this score and could indicate response to TPE imprecisely [14]. Accordingly the differences between our clinical (72% responders) and our EDSS (34% responders) response definitions reveal, that the accurate clinical response definition is better suitable for the treatment with TPE. Some limitations of our study have to be mentioned. The lack of a control group to compare our results with TPE and the retrospective character were limitations of our analysis. Other limitations were the lack of routine timing of treatment and of evaluation after TPE, heterogeneous treatment receiving groups and the unblinded assessment of the primary outcome. A prospective randomised trial performed to compare true and sham TPE in CIS and MS would be best to evaluate TPE effects and is warranted. As long as TPE represents the last therapeutical option for GCS-unresponsive patients, a clinical trial with patients assigned to a sham treatment cannot easily be justified due to the beneficial effects of TPE observed in available studies [14, 17, 22, 23, 25, 36, 37]. A randomisation of GCS-unresponsive patients into a cohort treated merely by GCS and patients additionally treated with TPE would end up in the same ethical dilemma. Although adverse events occurred in the minority of our patients, various risks of an extracorporeal treatment are further contraindications for sham TPE [38]. The relatively small number of TPE complications in our patients could be underestimated due to the retrospective assessment of adverse events. Bleeding complications through anticoagulation or adverse events through central venous puncture (e.g. thrombosis, infection or vascular damage) can occur and are unacceptable for sham treated patients at the current stage of experience with TPE in CIS and MS [38, 39]. To date, both the exact mechanisms of CIS and MS and the mechanism of the influence of TPE on these diseases are still not clear and need further research [40, 41]. In contrast to the disease activity of CIS and MS localised in the CNS, the depletion of inflammatory factors with TPE in plasma is achieved in the peripheral immune system [19, 40]. Therefore, effects of TPE are supposed to result from a breakdown of supply of immunologic factors maintaining CNS inflammation [14, 24, 40]. Pure elimination of autoantibodies and inflammatory mediators cannot explain all effects of TPE [19]. A direct impact on immune cells in MS and CIS is discussed [19, 40, 42]. In 2005, Keegan et al reported on favourable responses to TPE in relation to immunopathologic pattern II (antibody/complement-associated demyelination) and treatment failure in patients with pattern I and III [43]. These findings supported the hypothesis of humoral mechanisms of action in MS and TPE and should explain responses to TPE more easily. Complementary to Keegan’s observations, heterogeneous patterns of demyelination and different stages of damaged brain tissue are meanwhile supposed to respond differently to TPE and GCS [32, 43, 44]. The different responses to TPE-rates among the various MS types underline our deficits to explain effects of TPE in these patients. In the present study response to TPE-rates varied significantly between relapsing forms of disease (CIS, RR-MS) and progressive disease courses (SP-MS, PP-MS). An underlying amplifying immunologic process and irreversible axonal damage in the course of MS are discussed in these cases [45]. This may result in the chronification of the disease and could explain the worse response to TPE in SP-MS and PP-MS, but on the other hand the better results of TPE in CIS and RR-MS patients [45, 46].

Response to TPE was significantly more common in patients without the administration of DMD in our study. It can be hypothesized, that mechanisms of TPE may be more effective in an immune system only influenced by GCS. DMD interfere with various immune system components and could reduce the effectiveness of TPE in these patients. Certainly, no DMD was found predominantly in CIS and RR-MS patients with a greater response to TPE. We were unable to differentiate the impact of TPE with or without DMD and of different MS types in our study, as SP-MS and PP-MS patients almost all received DMD.

Beneficial TPE was significantly associated with a baseline EDSS ≤ 5.0 in the present study and goes along with observations from Magana and Keegan et al [15, 22]. EDSS values of > 5.0 were observed predominantly in the SP-MS and PP-MS group in our study and represented mainly chronic progressive patients with severe neurologic impairment. Neurodegeneration might predominate neuroinflammation and could explain the worse response to TPE in these patients. Consistent with previous studies in chronic progressive MS, beneficial effects of TPE were significantly fewer in SP-MS and PP-MS patients [47].

Interestingly, the time from relapse onset to the start of TPE did not influence the response rate significantly. In the majority of studies, time to treatment within 6 weeks was considered most relevant for treatment success [15, 22, 26]. Median time to TPE was around 8 weeks in our cohort, but beneficial effects of TPE were demonstrated in about 72% of patients. Axonal damage and irreversible neuronal impairment are known to develop most likely during prolonged CNS inflammation [22]. Therefore, a short time to treatment should be pursued to interrupt inflammation and response to TPE is achievable even after longer periods [14, 22]. Especially in patients with a severe neurological deficit, TPE should be considered independently from the duration of symptoms [14, 25]. The inflammatory intensity, the extent of Gd^+^ MRI lesions and individual disease stages at the time of TPE might influence the response to TPE more likely than a short time to treatment [14, 23, 25].

In line with observations from Magana et al successful TPE was related to Gd^+^ lesions in MRI in our study [15]. As Gd^+^ lesions represent blood brain barrier disruption and do reflect a state of acute CNS inflammation, mechanisms of TPE could be more effective in these patients [23]. Recently, Meca-Lallana et al investigated effects of TPE on radiologic resolution of ring-enhancing MS lesions in MRI [23]. In contrast to the importance of Gd^+^ lesions before TPE, the resolution of active lesions after TPE was not associated with response to TPE and did not correlate with the patients prognosis [23].

## Conclusion

In comparison to pre-existing small sample studies, response to TPE was verified in a larger cohort of 90 CIS and MS patients. Beneficial treatment effects in about 72% of patients and few adverse events, confirm the important role of TPE in the medical control of CIS and MS relapses. Gd^+^ lesions in MRI were the best predictor of the response to TPE in our study. TPE response was significantly more common in patients with relapsing (CIS, RR-MS) than in progressive disease courses (SP-MS, PP-MS). Other potential predictors of the response to TPE, as time from relapse onset, age, gender or the administered GCS dosage before TPE were independent from treatment success. Prospective clinical trials focusing on TPE in CIS and MS patients are encouraged.

## Supporting Information

S1 File Diagnostic findings, disease modifying drugs and univariate statistical analysis results. Diagnostic findings before the initiation of treatment **(Table A).** Administration of disease modifying drugs before the initiation of therapeutic plasma exchange (n = 47) **(Table B).** Univariate statistical analysis of therapeutic plasma exchange in clinically isolated syndrome and multiple sclerosis patients **(Table C).**
(PDF)
