# Supplementary material for: Response to Therapeutic Plasma Exchange as a Rescue Treatment in Clinically Isolated Syndromes and Acute Worsening of Multiple Sclerosis: A Retrospective Analysis of 90 Patients
Source: PLoS One. 2015 Aug 5;10(8):e0134583. doi: 10.1371/journal.pone.0134583 (PMC4526633; doi:10.1371/journal.pone.0134583)
Supplement: S1 File — Diagnostic findings before the initiation of treatment (Table A). Administration of disease modifying drugs before the initiation of therapeutic plasma exchange (n = 47) (Table B). Univariate statistical analysis of therapeutic plasma exchange in clinically isolated syndrome and multiple sclerosis patients (Table C). (PDF) [file pone.0134583.s001.pdf]

Table A in S1 File: Diagnostic findings before the initiation of treatment

	T_2 lesions in MRI (%)		Gd^+ lesions in MRI (%)		Pathologic MEP	Pathologic SSEP	Pathologic VEP
	brain	spinal	brain	spinal			
**CIS (n=21)**	71.4	65.0	52.4	33.3	10 of 16	10 of 18	16 of 17
**RR-MS (n=46)**	100	80.0	52.2	28.6	18 of 24	23 of 29	18 of 29
**SP-MS (n=18)**	100	100	77.8	22.2	10 of 11	9 of 9	8 of 8
**PP-MS (n=5)**	100	100	20.0	20.0	5 of 5	5 of 5	5 of 5
**Total (n=90)**	77.8	61.1	44.4	20.2	43 of 56	53 of 60	41 of 60

CIS = clinically isolated syndrome, Gd^+ = gadolinium contrast medium enhancement, MEP = motor-evoked potentials, MRI = magnetic resonance imaging, MS = multiple sclerosis, PP-MS = primary progressive MS with acute worsening, RR-MS = relapsing-remitting MS, SP-MS = secondary progressive MS with superimposed relapse, SSEP = somatosensory-evoked potentials, VEP = visual-evoked potentials, T_2 = T_2 weighted imaging. Pathologic result of MEP/SSEP/VEP = loss of potential or prolonged latency or definite site difference

Table B in S1 File: Administration of disease modifying drugs before the initiation of therapeutic plasma exchange (n=47)

**Disease modifying drug**	**N**
Interferone beta 1-a	12
Interferone beta 1-b	3
Intravenous immunglobuline	3
Mitoxantrone	8
Azathioprine	2
Natalizumab	3
Intermittent GCS treatment	8
Glatirameracetate	7
Cyclophosphamide	1

GCS = Glucocorticosteroid

Table C in S1 File: Univariate statistical analysis of therapeutic plasma exchange in clinically isolated syndrome and multiple sclerosis patients

Variable	TPE response (n)	TPE non-response (n)	Total (n)	*p*
**Group analysis**				
CIS vs. RR-MS	17 vs. 38	4 vs. 8	21/46	1.000
CIS vs. SP-MS	17 vs.7	4 vs. 11	21/18	**0.010**
RR-MS vs. SP-MS	38 vs. 7	8 vs. 11	46/18	**0.002**
CIS/RR-MS vs. SP-MS/PP-MS	55 vs. 10	12 vs. 13	67/23	**0.001**
**Gender**				0.451
Male	22	6	28	
Female	43	19	62	
**Age in years (median, range)**	35.0 (18-69)	41.0 (20-66)		0.057
**DMD**				
Yes	28	18	46	
No	37	7	44	**0.017**
**Disease activity before TPE**				0.488
0 relapses	17	4	21	

1 relapse<br>≥ 2 relapses	1<br>47	1<br>20	2<br>67	
**Worsening during GCS treatment**<br>Yes<br>No	 16<br>49	 12<br>13	 28<br>62	**0.043**
**Time to TPE**<br>≤ 6 weeks<br>> 6 weeks	 18<br>47	 7<br>18	 25<br>65	1.000
**Symptom extent before TPE**<br>Monosymptomatic<br>Polysymptomatic	 9<br>56	 6<br>19	 15<br>75	0.343
**GCS treatment before TPE**<br>1g MP only<br>1g and 2g MP	 16<br>44	 10<br>11	 26<br>55	0.104
**MEP before TPE**<br>Pathologic<br>Physiologic	 28<br>12	 15<br>1	 43<br>13	0.082

**VEP before TPE**				0.346
Pathologic	29	12	41	
Physiologic	16	3	19	
**SSEP before TPE**				0.173
Pathologic	37	16	53	
Physiologic	7	0	7	
**T_2 lesions in MRI**				1.000
Detected	57	19	76	
Not detected	1	0	1	
**Gd^+ lesions in MRI**				
Detected	42	5	47	**0.001**
Not detected	16	14	30	
**EDSS before TPE**				
EDSS ≤ 5	46	10	56	**0.014**
EDSS > 5	19	15	34	

CIS = Clinically Isolated Syndrome, DMD = Disease modifying drug, EDSS = Expanded disability status scale, Gd^+ = Gadolineum contrast medium enhancement, GCS = Glucocorticosteroid, MEP = Motor-evoked potentials, Monosymptomatic = single functional system affected, MRI = Magnetic resonance imaging, MS = Multiple sclerosis, Polysymptomatic = ≥ 2 functional systems affected, PP-MS = Primary

progressive MS with acute worsening, RR-MS = Relapsing-remitting MS, SP-MS = Secondary progressive MS with superimposed relapse, SSEP = Somatosensory-evoked potentials, T_2 = T_2 weighted imaging, TPE = Therapeutic plasma exchange, VEP = Visual-evoked potentials, vs. = versus. Pathologic result of MEP/SSEP/VEP = loss of potential or prolonged latency or definite site difference.

PP-MS patients excluded due to low patient number of this group.
